# Identification and physical characterization of a spontaneous mutation of the tobacco mosaic virus in the laboratory environment

**DOI:** 10.1038/s41598-021-94561-2

**Published:** 2021-07-23

**Authors:** Jenica L. Lumata, Darby Ball, Arezoo Shahrivarkevishahi, Michael A. Luzuriaga, Fabian C. Herbert, Olivia Brohlin, Hamilton Lee, Laurel M. Hagge, Sheena D’Arcy, Jeremiah J. Gassensmith

**Affiliations:** 1 Department of Chemistry and Biochemistry, The University of Texas at Dallas, 800 West Campbell Road, Richardson, TX 75080 USA; 2 Department of Bioengineering, The University of Texas at Dallas, 800 West Campbell Road, Richardson, TX 75080 USA

**Keywords:** Biochemistry, Proteomics, Proteins, Viral proteins

## Abstract

Virus-like particles are an emerging class of nano-biotechnology with the Tobacco Mosaic Virus (TMV) having found a wide range of applications in imaging, drug delivery, and vaccine development. TMV is typically produced *in planta*, and, as an RNA virus, is highly susceptible to natural mutation that may impact its properties. Over the course of 2 years, from 2018 until 2020, our laboratory followed a spontaneous point mutation in the TMV coat protein—first observed as a 30 Da difference in electrospray ionization mass spectrometry (ESI–MS). The mutation would have been difficult to notice by electrophoretic mobility in agarose or SDS-PAGE and does not alter viral morphology as assessed by transmission electron microscopy. The mutation responsible for the 30 Da difference between the wild-type (wTMV) and mutant (mTMV) coat proteins was identified by a bottom-up proteomic approach as a change from glycine to serine at position 155 based on collision-induced dissociation data. Since residue 155 is located on the outer surface of the TMV rod, it is feasible that the mutation alters TMV surface chemistry. However, enzyme-linked immunosorbent assays found no difference in binding between mTMV and wTMV. Functionalization of a nearby residue, tyrosine 139, with diazonium salt, also appears unaffected. Overall, this study highlights the necessity of standard workflows to quality-control viral stocks. We suggest that ESI–MS is a straightforward and low-cost way to identify emerging mutants in coat proteins.

## Introduction

Biotechnology based on non-infectious viral nanoparticles has been an emerging topic of research for the last two decades, with considerable advancement toward biomedical translation occurring recently^1, 2^. Nature has provided ample source material—viruses that infect humans, bacteria, and plants all fair game when engineering a new and more efficacious drug delivery, vaccine, and imaging platform. Plant-based viruses have gained attention in the field of biotechnology for several reasons. In addition to being robust, at least insofar as proteinaceous materials go, they have the benefit of being literally farmable—the viruses can be produced in plant tissue in a scalable approach. For instance, Medicago Inc, a biotechnology company presently based in Quebec City, has begun phase II–III trials of a plant-produced vaccine against COVID-19^3, 4^.

Likely the most studied virus in plant-harvested viral-nanotechnology is the Tobacco Mosaic Virus (TMV), a noninfectious, plant-based virus-like particle^2, 5–8^. It is rigid, monodisperse, thermostable, functionalizable^2, 6, 8^, and biocompatible^9–11^, giving it a wide range of applications including as a scaffold for biomolecules and small molecules, nanocontainers for drug delivery^5, 9, 12–19^, and as a platform for vaccine development and testing^10, 11, 20–23^.

TMV is a positive-sense single-stranded RNA virus under the *Tobamovirus* genus. Its RNA encodes four proteins: two 126- and 183-kDa replicase proteins, a 30 kDa movement protein, and a 17.5 kDa coat protein^5, 24^. The intact TMV contains 2130 copies of the coat protein assembled into a helical rod with a length of 300 nm, an outer diameter of 18 nm, and a pore diameter of 4 nm. Both exterior and interior surfaces of this hollow rod can be modified on solvent-exposed amino acids^6, 24^. We, like most groups that produce plant-based viral nanoparticles, use an extremophile strain of the Australian plant *Nicotiana benthamiana* for large-scale production of TMV. It is the most widely used host for plant virus replication as it is very susceptible to infection. An RNA silencing gene in *N*. *benthamiana* (*NbRdRP1m*) is mutated and less active, contributing to the species’ susceptibility of RNA-virus infection^25–27^.

Single-stranded RNA viruses, like TMV, are very susceptible to mutation^28^. Though the mechanism is not established yet, Sanjuán *et al.* (2016) suggested that the genetic material in viruses with single-stranded genomes are more exposed to oxidative deamination and other chemical stress^28^. In fact, mutant populations are ubiquitous in plant RNA viruses and their presence is rarely considered significant until the mutant phenotype dominates^29^. RNA viruses, except Coronavirus, contain RNA polymerases that lack 3′ → 5′ exonuclease proofreading mechanisms, which makes them more prone to error than DNA viruses^28, 30^. One study showed that infecting tomato plants containing the *Tm-2* Gene with TMV strain Ltbl, resulted in point mutations in the 30 kDa movement protein^31^. TMV is well-known to be susceptible to environmental and evolutionary stressors and the emergence of mutants should not be surprising; however, there is little discussion in the literature of how coat protein mutations might alter the viruses’ physical properties vis-à-vis chemical functionalization and antibody binding. Best practices for monitoring TMV mutation and the impacts of TMV mutation on virus usability are not well established. Knowing what to look for, the relative time scale, and how to identify mutation in plant-sourced viruses would be helpful to the broader community.

Here, we report the emergence of a mutant strain of TMV over the course of two years in the absence of any purposeful chemical or environmental stressor. The strain contains a single point mutation in the coat protein and was identified by electrospray ionization mass spectrometry (ESI–MS) of TMV stocks archived from January 2018 to January 2020. Over this period, the mutant strain largely displaced the wild-type strain, suggesting a competitive advantage. While we were following best practices for purifying and testing isolated TMV at the time, we have since identified additional practices that should be followed to identify emerging mutants. In many assays, the mutant was indistinguishable from wild-type. This work highlights the need for standard workflows to quality-control viral stocks in the chemical and bioengineering laboratory, particularly if they are being considered for translational purposes.

## Result and discussion

Our TMV stock is grown in *N. benthamiana* eight weeks after germination while the plants are around 11.5 cm tall. *N. benthamiana* is grown in a purpose-built plant growth room with constant temperature (22 °C) and humidity (67%). TMV (15 mg) from a prior harvest is added to 1 g of silicon carbide as an abrasive and rubbed into the leaves of *N. benthamiana.* Once the leaves become discolored, two weeks after infection, the leaves are collected and stored at − 80 °C until the virus is extracted as previously described^12^.

A small amount of TMV is always set aside for the next infection. In the literature, the TMV stock is typically characterized by electrophoretic mobility (*e.g.* SDS-PAGE), transmission electron microscopy (TEM), size exclusion chromatography, and intact protein mass spectrometry^6, 9, 12, 14, 24^. RNA sequencing has been done in some cases^32, 33^ but is not routinely reported. If mutant TMV strains have been detected by sequencing, they have not been widely reported in the literature to date.

**Figure 1:**
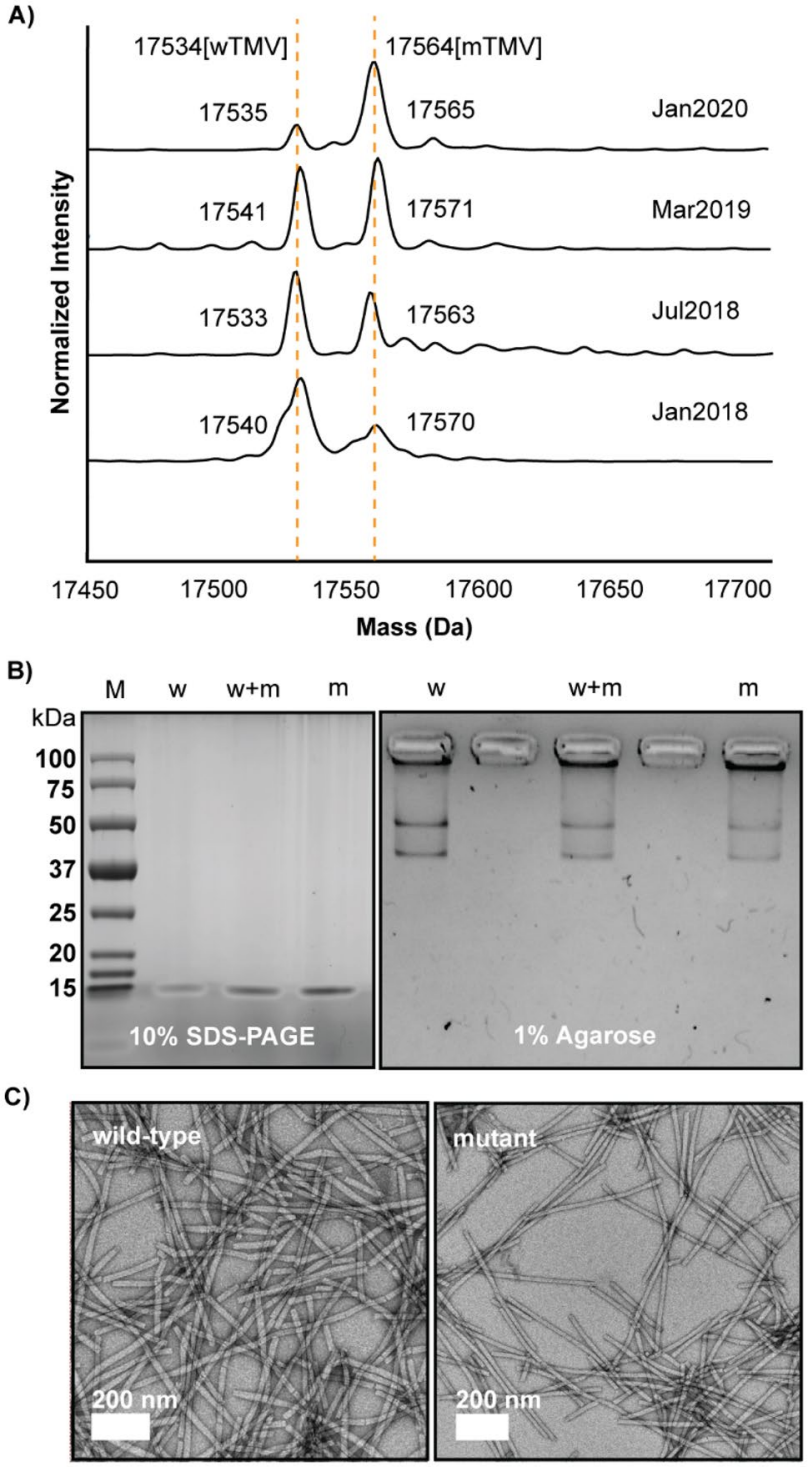
**(A)** Deconvoluted ESI–MS spectra of denatured TMV showing the emergence of a coat protein mutant from Jan 2018 till Jan 2020. The theoretical mass of wild-type TMV coat protein (wTMV) is 17,534 Da (including acetylation of the N-terminus). The mutant TMV coat protein (mTMV) is measuring approximately 30 Da larger. The relative abundance of the mTMV increased over time. **(B)** 10% SDS-PAGE and 1% agarose gel electrophoresis showing that the wTMV and mTMV migrate at the same rate. ‘w’ is mostly wTMV, ‘m’ is mostly mTMV, and ‘w + m’ is a mixture. **(C)** TEM micrographs of wTMV and mTMV showing that the rod-shaped viral morphology is not impacted by the mutation.

We monitor our TMV purifications using liquid chromatography mass spectrometry (LC–MS). We conducted LC–MS using ESI–MS on denatured viral samples. 20 µL of 10 mg/mL TMV was mixed with 40 µL of glacial acetic acid and the RNA removed via centrifugation. Over two years, from January 2018 till January 2020, we observed the appearance of a new mutant TMV strain (Fig. 1A). In Jan 2018, we observed the anticipated coat protein mass of 17,534 Da, the theoretical mass of wild-type TMV coat protein (wTMV) acetylated at its N-terminus^6, 9, 12, 34^. A second peak was also observed at approximately 17,564 Da. This peak was a relatively minor peak but was consistent in every sample of extracted TMV. By Jan 2020, the peak at 17,564 Da, which we now know is a mutant TMV coat protein (mTMV), had become dominant.

Aside from its mass, the mTMV is indistinguishable from wTMV. Characterization approaches that follow best practice per the literature, do not indicate the heterogeneity of the TMV preparations. When the wTMV and mTMV samples are analyzed by electromobility in SDS-PAGE and agarose gels (Fig. 1B), for example, there is no difference in protein migration. SDS-PAGE shows the typical single protein mass. Agarose, which measures the migration of intact viral nanoparticles, can be affected by both changes in mass, as well as charge, and it also showed no difference between wTMV and mTMV. The intact viral nanoparticles of both mTMV and wTMV (Fig. 1C) also show identical rod morphology in TEM. Had we not routinely conducted ESI–MS analyses of our isolates, the mutant would have gone undetected. Matrix assisted laser desorption ionization-time of flight mass spectrometry (MALDI-MS) is widely used to measure protein mass. From our assessment of the literature, most mass analyses for TMV coat proteins are conducted via MALDI-MS, including many from our group^6, 14, 32, 35^. Nominal resolution MALDI is attractive because the instruments are low cost, sample preparation and analyses are straightforward, and the gentle ionization conveniently produces the + 1 *m/z* peak. However, nominal resolution MALDI-TOF typically lacks the resolving power to identify small changes in mass on a large protein^36^.

**Figure 2:**
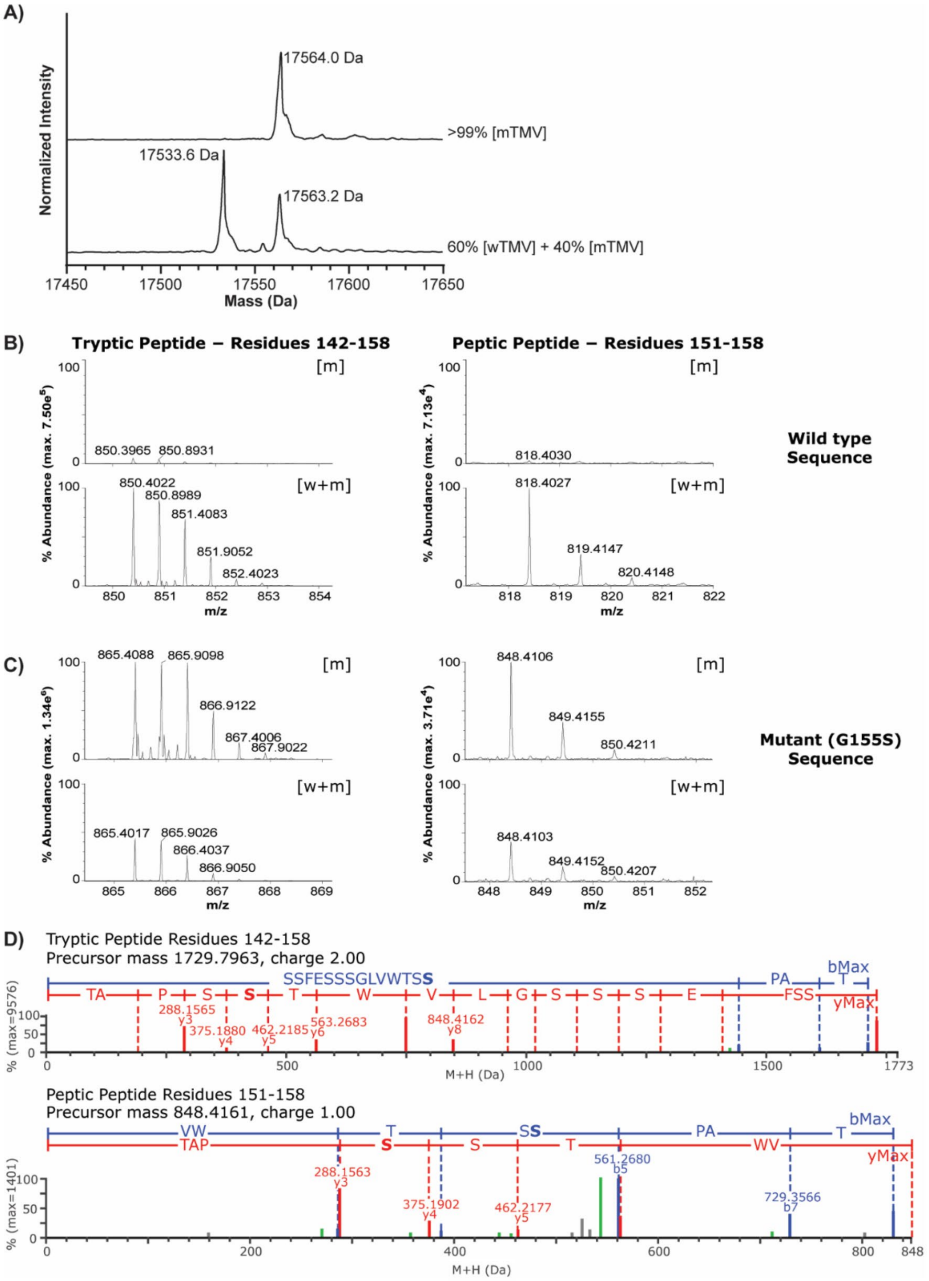
**(A)** Deconvoluted ESI–MS data of denatured TMV samples showing distribution between wTMV and mTMV. One sample contains 60% wTMV and 40% mTMV, while the other sample is almost entirely mTMV. **(B)** Peptide spectra recovered using the wild-type coat protein sequence. Peptide 142–158 (left) comes from a tryptic digest. Peptide 151–158 (right) comes from a peptic digest. Both peptides are reduced in the mTMV sample compared to the mixture of wTMV and mTMV. **(C)** Peptide spectra recovered using coat protein sequence containing S at position 155. Both peptides are more abundant in the mTMV sample compared to the mixture of wTMV and mTMV. **(D)** Fragmentation data for C-terminal peptides from the mTMV digests (top is trypsin; bottom is pepsin) when data was searched using a coat protein sequence containing the G155S mutation.

**Figure 3:**
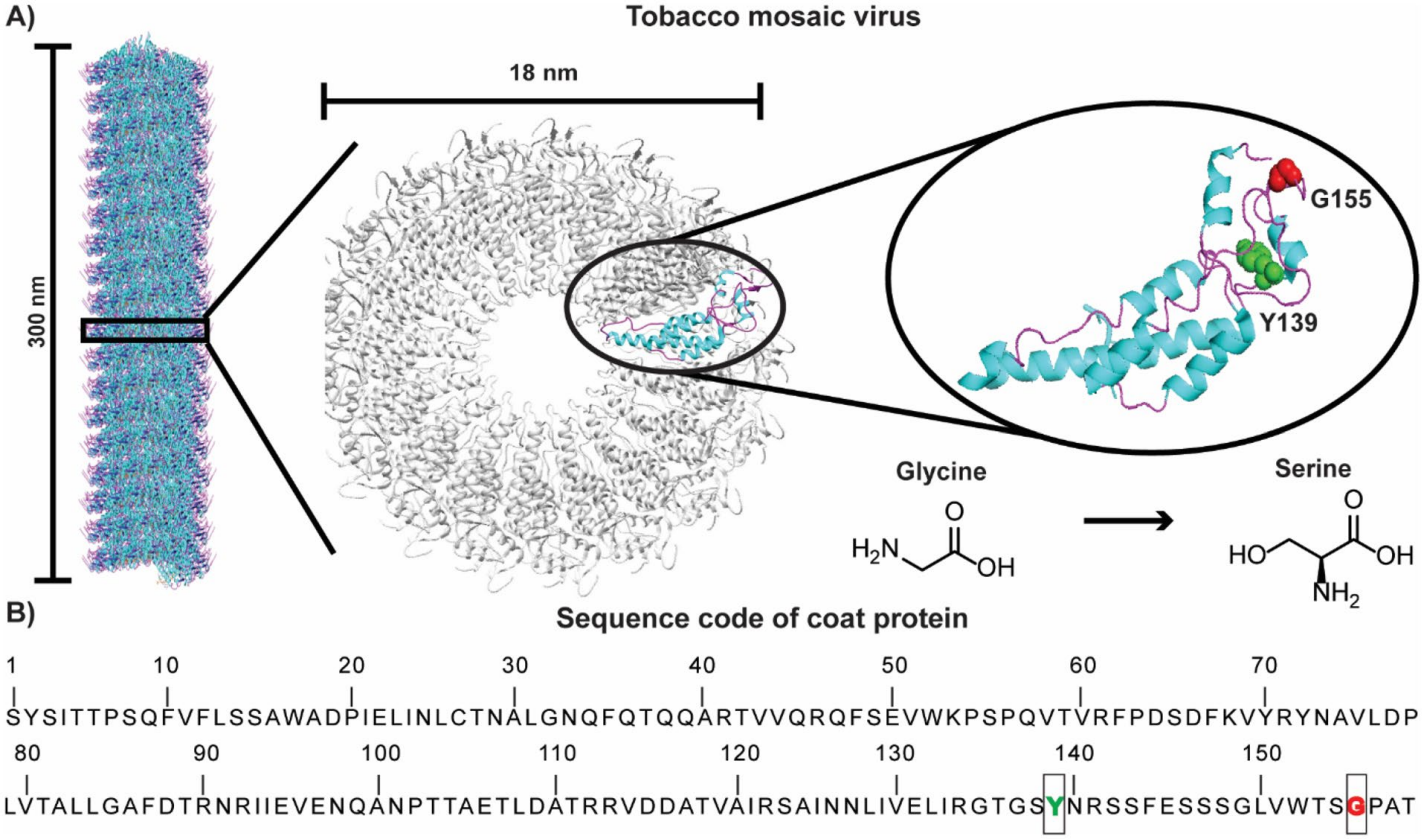
**(A)** TMV particle showing 300 nm in length and cross-section with an 18 nm diameter. A single coat protein is highlighted showing helices in cyan and loops in magenta. Gylcine 155 (red) and tyrosine 139 (green) are shown as spheres. Figure was prepared using PDB 3J06 in Chimera 1.15 (https://www.cgl.ucsf.edu/chimera/download.html) and PyMOL 2.5.1 (https://pymol.org/2/). **(B)** Coat protein sequence (https://www.rcsb.org/fasta/entry/3J06/display).

To identify the precise mutation(s) responsible for the 30 Da difference between wTMV and mTMV coat protein, we adopted a bottom-up approach. We compared a sample containing 60% wTMV and 40% mTMV, to one almost entirely composed of mTMV (Fig. 2A). Tryptic digests of both samples produced seven shared peptides that covered 90% of the coat protein sequence. Of these seven peptides, only one showed over an order of magnitude difference in intensity between the two samples (Fig. 2B left). This peptide covered residues 142–158, suggesting the TMV mutation was located within these C-terminal residues of the protein. For further confirmation, peptic digests of both samples were also performed. These similarly identified a change in intensity for a peptide covering residues 151–158 (Fig. 2B right). These data show that the mutation is in one of the last 8 residues of the coat protein (Fig. 3B).

We next generated a list of all possible single point mutations that would cause a mass increase of 30 ± 1 Da in the last 8 residues of the coat protein. The list contained nine possible sequences and each sequence was used to interrogate the tryptic and peptic digests of the mTMV sample. Only one sequence gave a passing result and identified the C-terminal peptide of the mTMV coat protein. This sequence contained a G155S mutation. In the two C-terminal peptides (142–158 and 151–158), collision-induced dissociation data show a serine at position 155 with consecutive fragmentation products recovered at the precise site of mutation (Fig. 2D). When using the sequence with G155S, the C-terminal peptides also had more intensity in the mTMV sample compared to the mixed sample (Fig. 2C). The difference between wTMV and mTMV is thus a G155S point mutation in the coat protein. At the nucleic acid level, the most likely mutation is a single guanine to adenine which changes the codon of residue 155 from GGU (glycine) to AGU (serine).

The mutation of glycine to serine at position 155 would not be expected to drastically impact the folding or stability of either the coat protein or the intact TMV. Glycine-155 is in a loop that is exposed to solvent on the exterior-side of the TMV rod (Fig. 3A). It is not involved in contacts between the many copies of the coat protein. A glycine to serine change is also somewhat conservative with both having small side chains. Differences may occur, however, as glycine allows for greater backbone flexibility and serine brings an additional hydroxyl group for hydrogen bonding. Algorithms predict a slight reduction in protein stability with the G155S mutation (ΔΔG = -0.41)^37^.

**Figure 4:**
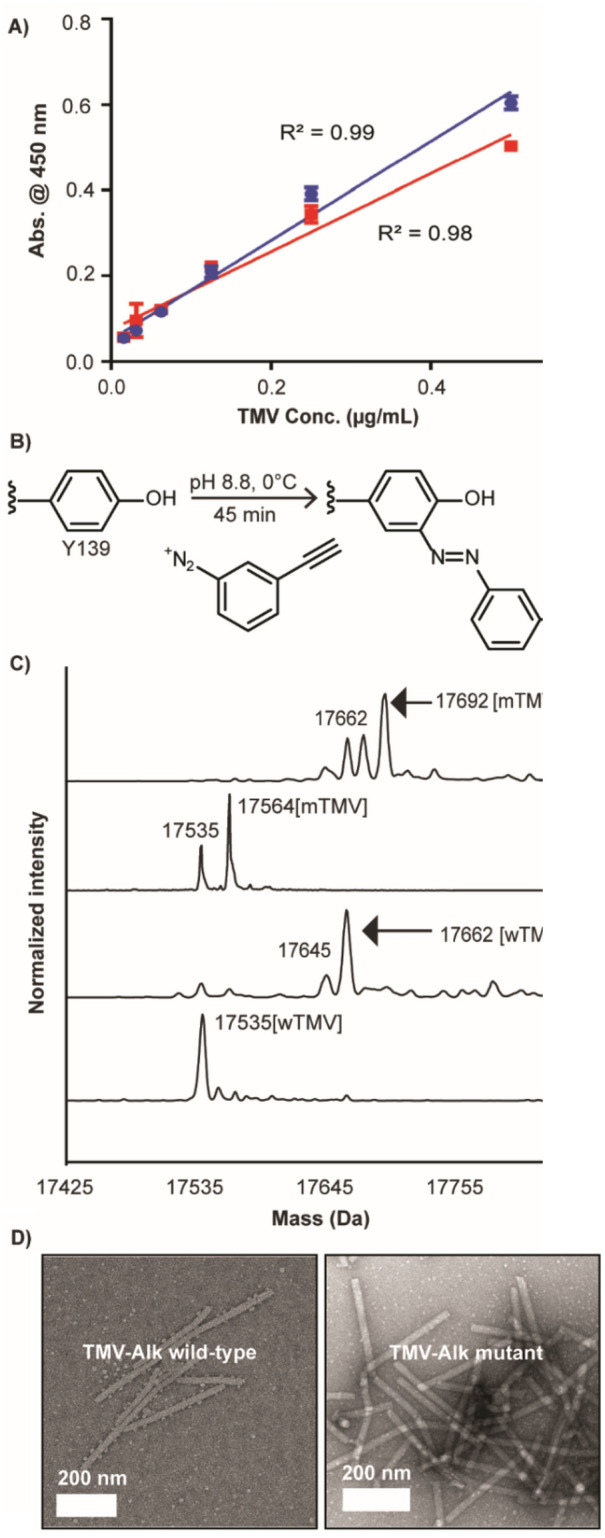
**(A)** ELISA showing that both wTMV (blue) and mTMV (red) have similar binding to polyclonal TMV antibodies (CAB 57400/1000). The correlation coefficient between the two curves is 0.998 using Pearson correlation coefficient, suggesting that there is no difference in antibody binding. The error bars show ± 2 standard deviations with a sample size n = 3. **(B)** Schematic showing the conjugation of diazonium salt with alkyne handle via diazonium coupling on the Y139. Conjugation should increase coat protein mass by 128 Da. This figure was made using Chemdraw 20.0 (https://perkinelmerinformatics.com/products/research/chemdraw/). **(C)** Deconvoluted ESI–MS spectra of TMV-Alkyne from both predominantly wTMV and mTMV. The wTMV-Alk shows a peak at 17,662 Da and a preceding small peak at 17,645 Da. The mTMV-Alk generates four peaks at 17,645 Da, 17,662 Da, 17,675 Da, and 17,692 Da. The first two peaks from the left, 17,645 Da and 17,662 Da, belong to wTMV-Alk. The last two peaks, 17,675 Da and 17,692 Da, belong to mTMV-Alk. **(D)** TEM showing that the morphology of TMV rods with alkyne remain intact. These results suggest that tyrosine functionalization is unaffected by this mutation.

Given the surface localization of the G155S mutation, we sought to test its impact on TMV antibody binding and chemical modification. With identical concentrations of TMV, changes in the relative binding of polyclonal antibodies would be obvious by comparing a dilution series in an enzyme-linked immunosorbent assay (ELISA). ELISA run on both mTMV and wTMV bind TMV antibodies at the same concentrations to produce linear curves with a correlation coefficient of 0.998, indicating the binding is almost identical (Fig. 4A). Therefore, it seems unlikely this mutation would have impacted any immunological or cell studies. So far, the ELISA result shows no significant difference between the binding of the epitopes of mutant and wild-type TMVs to TMV antibodies, but if there is any change in the epitope, it might likely be missed by the method, and further study is needed. TMV is also widely used for its facile chemical modifiability at the external tyrosine residue via a diazonium coupling reaction (Fig. 4B). We were able to show that the tyrosine residue can be functionalized through diazonium coupling quantitatively on the mTMV. Figure 4C shows the deconvoluted ESI mass spectra of wTMV-Alk at 17,662 Da—which is a 128 Da increase from wTMV mass from the attached azo-alkyne, although very small wTMV peak can still be observed on the spectrum. A preceding peak can be also observed (17,645 Da), which has been previously reported^6, 9^. For mTMV-Alk, a complete conjugation is observed with the major peak at 17,692 Da, a 30 Da increase from wTMV-Alk. The presence of wTMV in the m TMV sample, generated four peaks in total. Based on these data, we conclude that the mutation does not have an obvious effect on the ability to functionalize Y130 via diazonium coupling. The morphology between wTMV-Alk and mTMV-Alk remains the same (Fig. 4D). These data show that the G155S mutation had no obvious effects on the physical properties of the TMV.

## Conclusion

In a span of 2 years, the wild-type TMV has spontaneously mutated in the laboratory environment as observed in the ESI–MS data collected in that period. It should be noted that similar data collected on intact proteins using nominal resolution MALDI-TOF, where the detected ion is the + 1 *m/z*, would likely have insufficient resolution to detect the presence of single point mutant strains. Further RNA sequencing methods that can detect mutants when they are not the dominant species are complicated and expensive. Bottom-up analysis and peptide sequencing with collision-induced dissociation identified the residue 155 as the site of a glycine to serine point mutation. Dramatic physical effects of this mutation are not predicted but given its surface localization there was the possibility of altered binding and surface chemistry. However, ELISA shows that both mutant and wild-type TMV have identical binding to TMV antibodies and can be equally functionalized at tyrosine 139. It is unclear precisely why the G155S mutant emerged and overtook the wild-type in our laboratory environment. Our discovery, however, highlights the necessity to monitor every batch of purified TMV as spontaneous point mutation can occur even in the absence of stress and mutagens.

## Methods

### Materials

Chemicals were purchased from Sigma-Aldrich (St. Louis, MO), ThermoFisher Scientific (Pittsburgh, PA), Agdia (Elkhart, IN), ChemImpex (Wood Dale, IL), Alfa Aesar (Ward Hill, MA), and TCI America (Portland, OR) and were used without further purification.

### Instrumentation

LC–MS were obtained using an Agilent 1100 HPLC with a PLRP-S column for separation and an AB Sciex 4000 QTRAP system for detection; also, a Waters SYNAPT G2-Si Q-TOF with an M-class UPLC. TEM was conducted using a JEOL JEM-1400Plus transmission electron microscope. Bio-rad ChemiDoc MP was used for gel imaging. ELISA data were obtained using a BioTek Synergy H4 Hybrid microplate reader.

### Expression of TMV

The tobacco (*Nicotiana benthamiana)* plants were grown for 8 weeks and infected with TMV solution with 2 weeks of incubation. The harvested infected leaves were stored at − 80 °C. About 100 g of leaves were blended with cold (4 °C) potassium phosphate (KP) buffer (0.1 M, 1000 mL, pH 7.4) and 2-mercaptoethanol (0.2% (v/v)) was added. It was subsequently ground to have an effective extraction. The slurry was filtered and centrifuged at 11,000 × *g* (4 °C, 20 min). The resulting supernatant was filtered again. The volume was measured and an equal amount of chloroform/1-butanol with 1-to-1 ratio was mixed (4 °C, 30 min). Another centrifugation was done at 4500 × *g* for 10 min. The collected aqueous phase was mixed with NaCl (final concentration of 0.2 M), PEG 8000 (8% (w/w)), and Triton X-100 surfactant (1% (w/w)). It was stirred on ice for 30 min followed by storing for 1 h at 4 °C. It was centrifuged again at 22,000 × *g* (4 °C, 15 min). The pellet was collected and resuspended in KP buffer (0.1 M, pH 7.4) and was stored at 4 °C overnight. The suspension was added carefully to 40% (w/v) sucrose gradient in KP buffer (0.01 M, pH 7.4) and centrifuged using swing bucket rotor at 96,000 × *g* for 2 h. The blue band was collected with the assistance of LED light shined upward from the bottom of the centrifuge tube. The colloidal suspension from blue band was centrifuged at 360,562 × *g* for 1.5 h. The pellet was resuspended in KP buffer (0.01 M, pH 7.4).

### Transmission electron microscopy (TEM)

TEM imaging was performed on a JEOL JEM-1400 + transmission electron microscope. Samples were prepared by incubating 5 µL of ~ 0.1 mg/mL TMV in water on a 300 mesh formvar-coated copper grid for 30 s. The sample was then stained with 5 μL 2% uranyl acetate for an additional 30 s. The excess liquid was wicked away with a Whatman (#1) filter paper and the grids were left to air dry. Images were taken with an accelerating voltage of 120 kV.

### ESI–MS

TMV samples were prepared by denaturing 20 µL of 10 mg/mL in 40 µL of glacial acetic acid. Sample was then centrifuged at 4300 × *g* for 10 s to separate the precipitated RNA. The supernatant was collected and run on an Agilent 1100 series HPLC system with a PLRP-S column for separation followed by a 4000 QTRAP mass spectrometer. The flow rate is 0.250 mL/min, and the solvent system comprises of Milli Q water, 0.1% formic acid, and pH 7.0 in a 0.1 M sodium phosphate buffer. This system was used for running both TMV and TMV-Alk samples.

### Electrophoretic mobility assays

1% (w/v) Agarose gels were used. The sample was prepared by mixing 3 µg TMV with 5 µL Thermo Scientific 6× DNA Loading Dye. From that mixture, 4 µL was added to each well. The gel was run at 100 eV for 45 min, stained with coomassie brilliant blue, and visualized using Bio-rad ChemiDoc MP gel imager.

10% SDS-PAGE gel was used. The sample was prepared by mixing 3 µg TMV with 5 µL of SDS loading dye (β-Mercaptoethanol (5%), Bromophenol blue (0.02%), Glycerol (30%), SDS (Sodium dodecyl sulfate 10%), Tris–Cl (250 mM, pH 6.8)) and 5 µL of 0.1 M dithiothreitol. The mixture was boiled for 10 min. 4 µL of sample was added to each well and the gel was run at 100 eV for 45 min, stained with coomassie brilliant blue, and visualized using Bio-rad ChemiDoc MP gel imager.

### Bottom-up proteomics

#### Digestion

Trypsin digestion was performed according to manufacturer’s protocol (Thermo Scientific, Product No. 90057). Briefly, TMV samples were dialyzed into a solution of 8 M urea and 50 mM triethylammonium bicarbonate (TEAB) at pH 8.5. 500 mM DTT was added to the sample to a final concentration of 20 mM and incubated for 1 h at 60 °C. 1 M Iodoacetamide was prepared and added to the sample to a final concentration of 40 mM and incubated for 30 min at room temperature in darkness. The alkylation reaction was quenched by adding 500 mM DTT solution to a final concentration of 10 mM. The samples were then diluted to reduce the concentration of urea to 1 M by adding 50 mM TEAB, pH 8.5. Trypsin in 50 mM TEAB pH 8.5 was added to the samples to a final protease-to-protein ratio of 1:20 (w/w) and incubated for 24 h at 37 °C. Samples were then flash-frozen and stored until injection. Samples for pepsin digestion were diluted to 1 µM in TEAB pH 8.5. Samples were then mixed 1:1 (v/v) with a solution of 1.6 M GuHCl, 0.8% formic acid at pH 2.3 to prepare them for on-line pepsin digestion. Samples were then flash-frozen and stored until injection.

#### LC–MS

LC–MS (data presented in Fig. 2) was performed using a Waters HDX manager and SYNAPT G2-Si Q-TOF. Two technical replicates of each sample were analyzed. Pepsin digest samples were digested on-line using *Sus scrofa* pepsin A (Waters Enzymate BEH) at 15 °C. Peptides were desalted on a C18 pre-column (ACQUITY UPLC BEH C18 VanGuard Pre-column) for 3 min at 100 μl min^−1^ and 1 °C. The liquid-chromatography buffer was 0.1% formic acid. Peptides were separated over a C18 column (Waters Acquity UPLC BEH) and eluted with a linear 3–40% (v/v) acetonitrile gradient for 7 min at 40 μl min^−1^ and 1 °C.

Mass-spectrometry data were acquired using positive-ion mode in HDMS^E^ mode, collecting both low-energy (6 V) and high-energy (ramping 22–44 V) peptide-fragmentation data for peptide identification. All samples were acquired in resolution mode. Capillary voltage was set to 2.8 kV for the sample sprayer. Desolvation gas was set to 650 l h^−1^ at 175 °C. The source temperature was set to 80 °C. Cone and nebulizer gas was flowed at 90 l h^−1^ and 6.5 bar, respectively. The sampling cone and source offset were both set to 30 V. Data were acquired at a scan time of 0.4 s with a m/z range of 100–2000. Mass correction was done using [Glu1]–fibrinopeptide B as a reference mass.

#### Data processing

Raw data were processed by PLGS (Waters Protein Lynx Global Server 3.0.2) using a database containing *Sus scrofa* pepsin A and native TMV coat protein. In PLGS, the minimum fragment ion matches per peptide was 3, and methionine oxidation and N-terminal acetylation were allowed. Trypsin samples included cysteine carbamidomethylation (CAM) as a fixed modifier. The low and elevated energy thresholds were 250 and 50 counts, respectively, and the overall intensity threshold was 750 counts. Peptides were curated in DynamX 3.0 with thresholds of 0.3 products per amino acid and one consecutive product.

### ELISA

96-well Microtiter Plates High Bind-Solid (ACC 00948/0005 Agdia) were coated with 100 µl of diluted (1:200) capture antibody rabbit anti-TMV IgG (CAB 57400/1000 TMV Capture antibody Agdia) in coating buffer (0.015 M Na_2_CO_3_, 0.034 M NaHCO_3_, NaN_3_ in dH_2_O, pH 9.6) and incubated overnight at 4 °C. The plate was washed 3 × with washing buffer (0.2% (v/v) Tween-20 in PBS, pH 7.4). After washing, wells were blocked with blocking buffer (1% (w/v) BSA in washing buffer, pH 7.4) at RT for 1 h, followed by 4 × washes with washing buffer. 100 μL of native TMV and mutant TMV—concentrations determined by Lowry assay (0.5–0.015 µg/ml)—were serially diluted with sample extraction buffer (0.009 M Na_2_SO_4_, 2% (v/v) Polyvinylpyrrolidone (PVP) 40 k, 0.2% (w/v) Powdered egg (chicken) albumin, 0.003 M NaN_3_, 0.2% (v/v) Tween-20 in washing buffer) was then added to each well and incubated at RT for 2 h. Wells were then washed 8 × with washing buffer, followed by the addition of 100 μL alkaline phosphatase-conjugated rabbit anti-TMV IgG in conjugate buffer (0.5 mg/mL BSA, 2% (v/v) PVP 40 k, and 0.003 M NaN_3_ in washing buffer) and incubated at RT for 2 h. Wells were then washed 8 × with washing buffer, then developed by adding 100 μL one-step *p*-nitrophenylphosphate (PNPP) substrate for 45 min at RT. The plate was read at 405 nm, 420 nm, and 450 nm, and the absorbance values of buffer blank wells averaged and subtracted from the entire plate. Experiments were performed in triplicate.

### TMV functionalization

The diazonium salt is prepared by carefully mixing 200 μL of 0.30 M p-toluenesulfonic acid monohydrate, 75 μL of 0.68 M 3-ethynylaniline, and 25 μL 3.0 M sodium nitrite followed by incubation in ice for 1 h without light exposure. The resulting diazonium salt (50 μL) was added to a 2 mg/mL wTMV or mTMV solution in 0.1 M borate buffer at pH 8.8. This was incubated on ice for 45 min. The resulting TMV-alkyne was purified and concentrated via centrifuge filtration using an EMD Millipore Amicon Ultra Centrifugal Filter Unit (10,000 MW Cutoff) (4303×*g*) (Suppl. Information).

### Regulatory and compliance

The authors comply with the IUCN Policy Statement on Research Involving Species at Risk of Extinction and the Convention on the Trade in Endangered Species of Wild Fauna and Flora. This plant and plant use studies were approved by the University of Texas at Dallas Institutional Biosafety and Chemical Safety Committee. Seeds and plants used are not listed as endangered or threatened and were a gift from Prof. James Culver at the University of Maryland.

## Supplementary Information

Supplementary Figure S1.
