# Supplementary material for: Identification and physical characterization of a spontaneous mutation of the tobacco mosaic virus in the laboratory environment
Source: Sci Rep. 2021 Jul 23;11:15109. doi: 10.1038/s41598-021-94561-2 (PMC8302582; doi:10.1038/s41598-021-94561-2)
Supplement: Supplementary file 1 — Supplementary Figure S1. [file 41598_2021_94561_MOESM1_ESM.docx]

***Supplementary Information***

Identification and Physical Characterization of a Spontaneous Mutation of the Tobacco Mosaic Virus in the Laboratory Environment

Jenica L. Lumata^1,+^, Darby Ball^1,+^, Arezoo Shahrivarkevishahi^1^, Michael A. Luzuriaga^1^, Fabian C. Herbert^1^, Olivia Brohlin^1^, Hamilton Lee^1^, Laurel M. Hagge^1^, Sheena D’Arcy^1,2,^* and Jeremiah J. Gassensmith^1,2,^*

^1^Department of Chemistry and Biochemistry, ^2^Department of Bioengineering, The University of Texas at Dallas, 800 West Campbell Road, Richardson, TX 75080, USA

^+^equal contribution

*Corresponding Authors emails:

sheena.darcy@utdallas.edu, Gassensmith@utdallas.edu


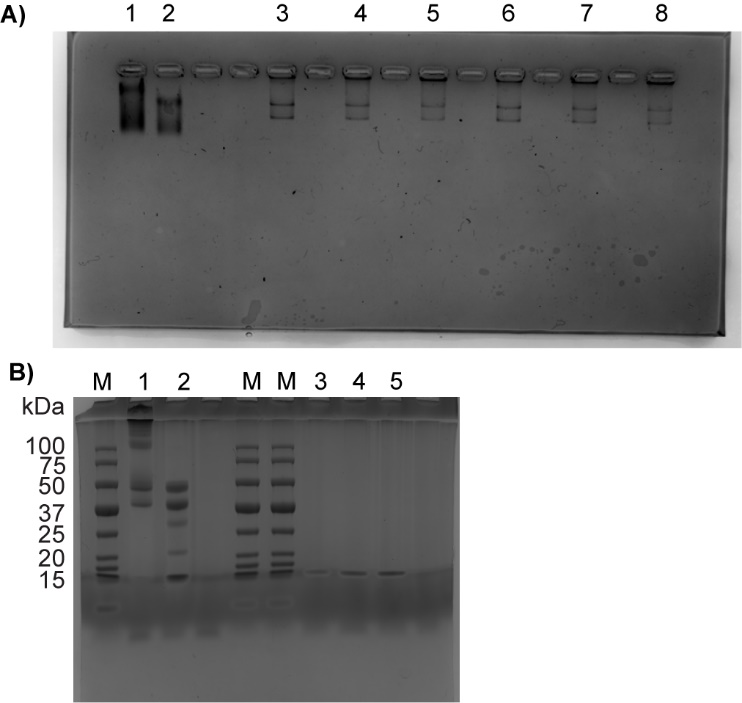


**Supplementary** Figure 1. Full Agarose and SDS gel images used portrayed in Fig. 1B. A) 1% Agarose stained with Coomasie brilliant blue. Sample identification: nonrelated samples (1 and 2), wild-type TMV (3), wild- and mutant TMV (4), mutant TMV (5), wild-type TMV (6), wild- and mutant TMV (7), and mutant TMV (8) at 0.2mg/mL. B) SDS gel with stained with Coomasie brilliant blue. Identification: marker (M), nonrelated samples (1 and 2), wild-type TMV (3), wild- and mutant TMV (4), mutant TMV (5) at 0.2mg/mL.
